# Supplementary material for: Regulation of inflammation and protection against invasive pneumococcal infection by the long pentraxin PTX3
Source: eLife. 2023 May 24;12:e78601. doi: 10.7554/eLife.78601 (PMC10266767; doi:10.7554/eLife.78601)
Supplement: Figure 8—source data 1. [file elife-78601-fig8-data1.zip › Figure 8 – Source Data 1c.pptx]

## Slide 1
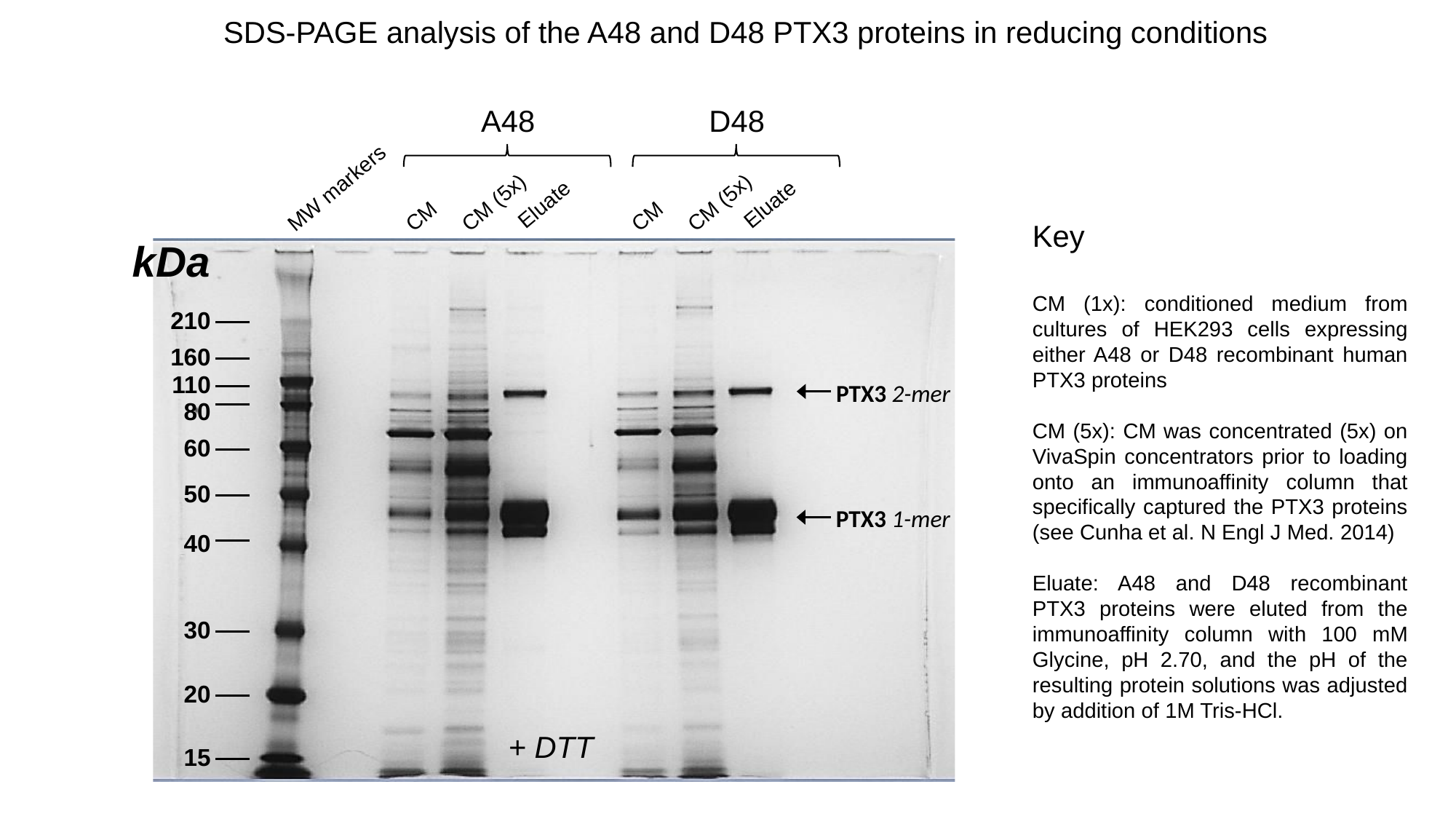

SDS-PAGE analysis of the A48 and D48 PTX3 proteins in reducing conditions
A48
D48
MW markers
Eluate
Eluate
CM (5x)
CM (5x)
CM
CM
Key
CM (1x): conditioned medium from cultures of HEK293 cells expressing either A48 or D48 recombinant human PTX3 proteins
CM (5x): CM was concentrated (5x) on VivaSpin concentrators prior to loading onto an immunoaffinity column that specifically captured the PTX3 proteins (see Cunha et al. N Engl J Med. 2014)
Eluate: A48 and D48 recombinant PTX3 proteins were eluted from the immunoaffinity column with 100 mM Glycine, pH 2.70, and the pH of the resulting protein solutions was adjusted by addition of 1M Tris-HCl.
kDa
210
160
110
PTX3 2-mer
80
60
50
PTX3 1-mer
40
30
20
+ DTT
15

## Slide 2
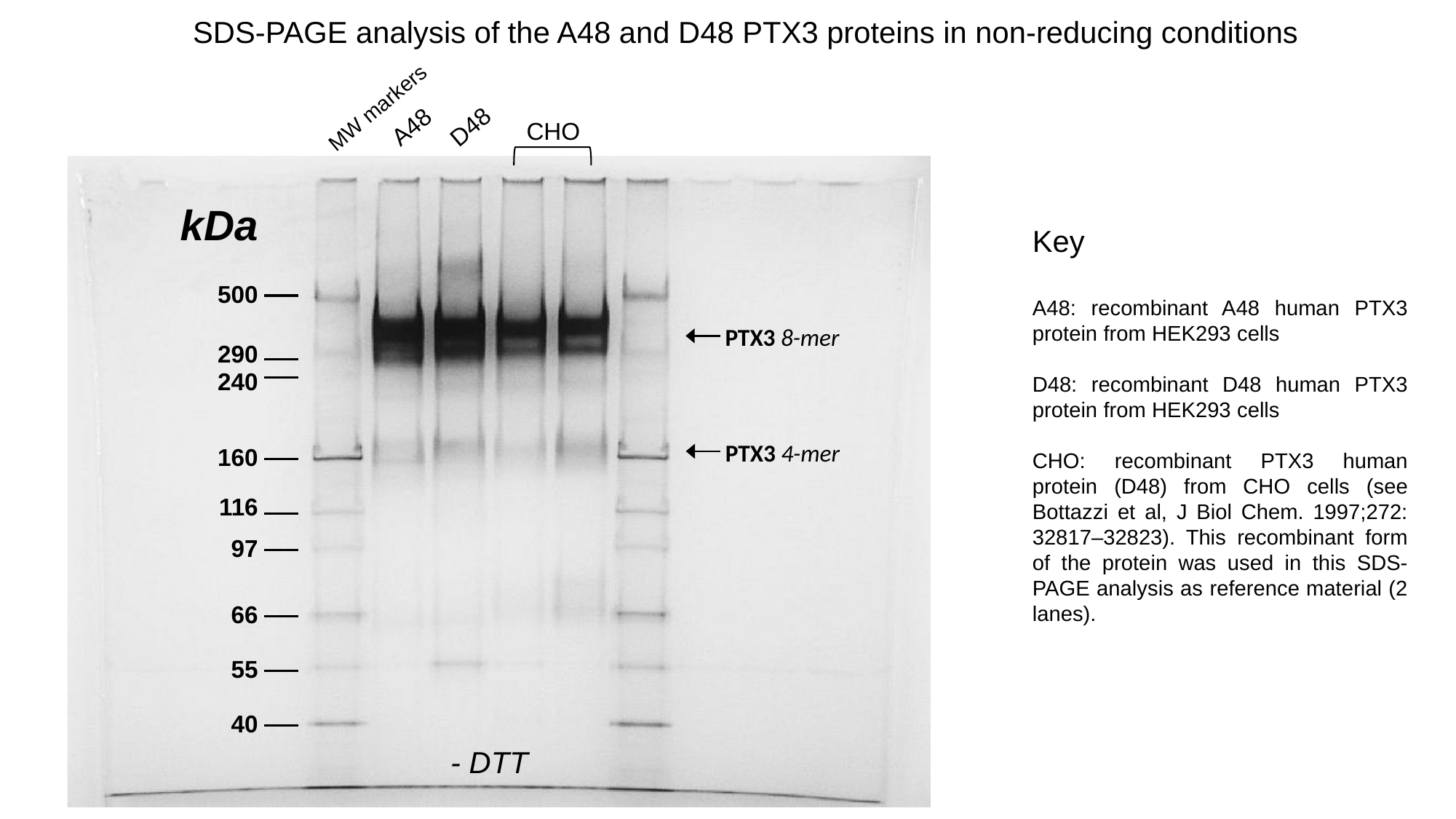

SDS-PAGE analysis of the A48 and D48 PTX3 proteins in non-reducing conditions
MW markers
A48
D48
CHO
kDa
Key
A48: recombinant A48 human PTX3 protein from HEK293 cells
D48: recombinant D48 human PTX3 protein from HEK293 cells
CHO: recombinant PTX3 human protein (D48) from CHO cells (see Bottazzi et al, J Biol Chem. 1997;272: 32817–32823). This recombinant form of the protein was used in this SDS-PAGE analysis as reference material (2 lanes).
500
PTX3 8-mer
290
240
PTX3 4-mer
160
116
97
66
55
40
- DTT
