# Supplementary figures and images for: Regulation of inflammation and protection against invasive pneumococcal infection by the long pentraxin PTX3
Source: eLife. 2023 May 24;12:e78601. doi: 10.7554/eLife.78601 (PMC10266767; doi:10.7554/eLife.78601)

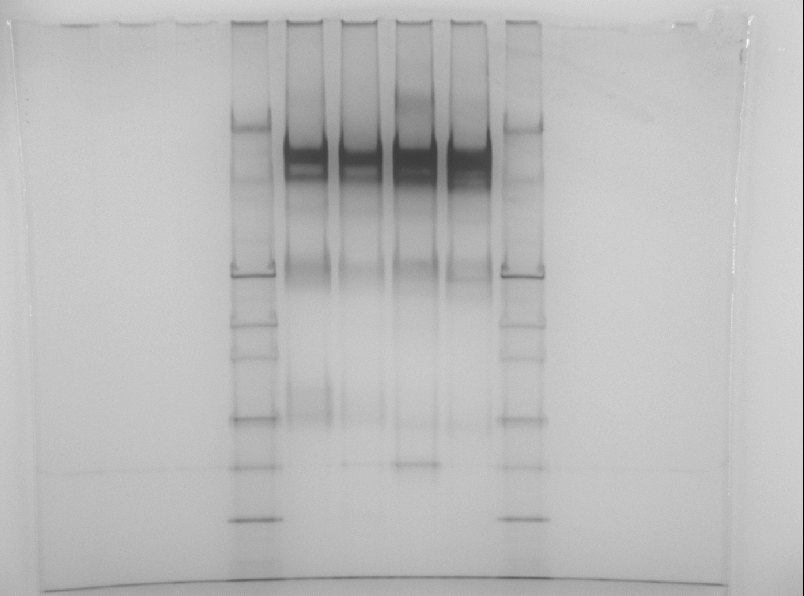

Supplement: Figure 8—source data 1. [file elife-78601-fig8-data1.zip › Figure 8 – Source Data 1a.jpg]

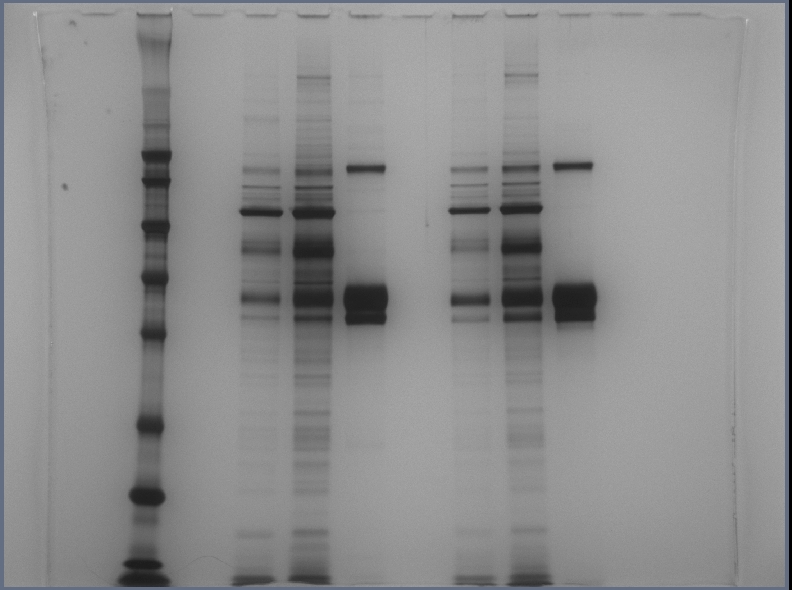

Supplement: Figure 8—source data 1. [file elife-78601-fig8-data1.zip › Figure 8 – Source Data 1b.jpg]
