# Supplementary material for: Regulation of inflammation and protection against invasive pneumococcal infection by the long pentraxin PTX3
Source: eLife. 2023 May 24;12:e78601. doi: 10.7554/eLife.78601 (PMC10266767; doi:10.7554/eLife.78601)
Supplement: MDAR checklist [file elife-78601-mdarchecklist1.pdf]

Westbrook Centre
Milton Road
Cambridge CB4 1YG, UK
P 01223 855340
W elifesciences.org
T @elife

Materials Design Analysis Reporting (MDAR) Checklist for Authors

The MDAR framework establishes a minimum set of requirements in transparent reporting mainly applicable to studies in the life sciences.

eLife asks authors to **provide detailed information within their article** to facilitate the interpretation and replication of their work. Authors can also upload supporting materials to comply with relevant reporting guidelines for health-related research (see EQUATOR Network), life science research (see the BioSharing Information Resource), or animal research (see the ARRIVE Guidelines and the STRANGE Framework; for details, see *eLife*'s Journal Policies). Where applicable, authors should refer to any relevant reporting standards materials in this form.

For all that apply, please note **where in the article** the information is provided. Please note that we also collect information about data availability and ethics in the submission form.

Materials:

Newly created materials	Indicate where provided: section/figure legend	N/A
GFP expressing *S. pneumoniae* serotype one is the only newly created material described	Materials And Methods section / Bacterial preparation line 624 to 632	

Antibodies	Indicate where provided: section/figure legend	N/A
anti-CD45-Brilliant Violet 605 (Biolegends 103155)<br>anti-CD11b APC-Cy7 (BD Bioscience 561039)<br>anti-SiglecF-eFluor 660 (Invitrogen 50-1702-80)<br>anti-Ly6C-FITC (BD Bioscience 561085)<br>anti-Ly6G-PE-CF594 (BD Bioscience 562700).	Materials And Methods section / Flow cytometry line 623 to 827	

DNA and RNA sequences	Indicate where provided: section/figure legend	N/A
Gapdh (Forward, 5'-GCAAAGTGGAGATTGTTGCCAT-3', Reverse, 5'-CCTTGACTGTGCCGTTGAATTT-3') and Ptx3 (Forward, 5'-CGAAATAGACAATGGACTCCATCC-3', Reverse, 5'-CAGGCGCACGGCGT-3')	Materials And Methods / Gene expression quantification by real-time RT-PCR line 786 and 788	

Cell materials	Indicate where provided: section/figure legend	N/A
Human Umbilical Vein Endothelial Cells (PromoCell C-12203) Murine lung capillary endothelial cell line (1G11) were created in our laboratory.	Materials And Methods / Cell culture and stimulation line 684 and 688	
Human neutrophils were prepared from fresh blood collected from volunteers in the IRCCS Humanitas Research Hospital. Volunteers were either males and females from 25 to 60 years old Caucasians.	Materials And Methods / Cell culture and stimulation line 690	

Experimental animals	Indicate where provided: section/figure legend	N/A
All mice used in this study were on a C57BL/6J genetic background. PTX3-deficient mice were generated as described in (Garlanda et al., 2002). Ptx3-/- and P-selectin (Selp-/-) double deficient mice were generated as described in (Doni et al., 2015). Csf3r-/- mice were generated as described in (Ponzetta et al., 2019). Wild-type (WT) mice were obtained from Charles River Laboratories (Calco, Italy) or were cohoused littermates of the gene-deficient mice used in the study. Ptx3-/-, Csfr3-/-, Ptx3loxP+/+Cdh5cre+/+, Ptx3loxP+/+Cdh5cre-/-, Selp-/-, Ptx3-/-Selp-/- and WT mice were bred and housed in individually ventilated cages in the SPF animal facility of Humanitas Clinical and Research Center or purchased from Charles River (Milan) and acclimated in the local animal facility for at least one weeks prior to infection.<br>C57BL/6J (Jax) Strain Code 632	Materials and Methods / Mice line 599 to 608	

Plants and microbes	Indicate where provided: section/figure legend	N/A
Streptococcus pneumoniae serotype 1 ST304<br>*Streptococcus pneumoniae* serotype 3 ATCC6303	Materials and Methods / Bacterial preparation line 619 to 620	

Design:

Experimental study design (statistics details) *		
**For in vivo studies: State whether and how the following have been done**	**Indicate where provided: section/figure legend. If it could have been done, but was not, write "not done"**	**N/A**
Sample size estimation was determined for each read-out by performing pilot experiments and determining the Cohen's effect size d (Lakens, 2013). Sample size were then estimated using G*Power software (version 3.1.9.7) to perform an a priori power analyses considering the d calculated as described above, an α error probability of 0.05 and 0.01 and a power level (1-β error probability) of 0.8 and considering the appropriated statistical analyses test (Faul et al., 2007).	Materials and Methods / Statistical analysis line 848 to 854	

Sample definition and in-laboratory replication	Indicate where provided: section/figure legend	N/A

Each experiment was replicated at least twice	indicated each time in figure legend	
Each replicate represent biological replicates		

**Ethics**	**Indicate where provided: section/submission form**	**N/A**
DNA was obtained from 57 pediatric patients with invasive pulmonary disease (IPD) and 521 age- and sex-matched healthy controls from the cohort described by Garcia-Laorden and collaborators (García-Laorden et al., 2020). Ethics Statement is described in García-Laorden et al., 2020.	Materials and Methods / Genotyping line 831 to 833.	
Procedures involving animals handling and care were conformed to protocols approved by the Humanitas Clinical and Research Center (Rozzano, Milan, Italy) in compliance with national (4D.L. N.116, G.U., suppl. 40, 18-2-1992 and N. 26, G.U. march 4, 2014) and international law and policies (European Economic Community Council Directive 2010/63/EU, OJ L 276/33, 22.09.2010; National Institutes of Health Guide for the Care and Use of Laboratory Animals, U.S. National Research Council, 2011). All efforts were made to minimize the number of animals used and their suffering. The study was approved by the Italian Ministry of Health (742/2016-PR)	Materials and Methods / Mice line 608 to 615.	

Analysis:

**Attrition**	**Indicate where provided: section/figure legend**	**N/A**
No exclusion criteria were applied to our data analysis		

**Statistics**	**Indicate where provided: section/figure legend**	**N/A**
Statistical differences were analyzed using the non-parametric Mann-Whitney test for two groups comparison, or the non-parametric Krukal-Wallis test with post-hoc corrected Dunn's test for multiple comparison of the mean with unequal sample size; survival analysis was performed with the logrank test with Mantel-Cox method.<br>All polymorphisms had a call rate of 100%, and were tested for Hardy-Weinberg equilibrium (HWE) in controls before inclusion in the analyses (P-HWE >0.05). In detail, deviations from HWE were tested using the exact test (Wigginton et al., 2005) implemented in the PLINK software. For each SNP, a standard case-control analysis using allelic chi-square test was used to provide asymptotic P values, odds ratio (OR), and 95% confidence interval (CI), always referring to the minor allele. Haplotype analysis and	Materials and Methods / Statistical analysis line 842 to 846 and line 856 to 864.	

phasing was performed considering either all three SNPs together or by using the sliding-window option offered by PLINK. All P values are presented as not corrected; however, in the relevant tables, Bonferroni-corrected thresholds for significance are indicated in the footnote.		

**Data availability**	**Indicate where provided: section/submission form**	**N/A**
All data generated or analysed during this study are included in the manuscript and supporting file		

Reporting:

The MDAR framework recommends adoption of discipline-specific guidelines, established and endorsed through community initiatives.

**Adherence to community standards**	**Indicate where provided: section/figure legend**	**N/A**
State if relevant guidelines (e.g., ICMJE, MIBBI, ARRIVE, STRANGE) have been followed, and whether a checklist (e.g., CONSORT, PRISMA, ARRIVE) is provided with the manuscript.		

---

* We provide the following guidance regarding transparent reporting and statistics; we also refer authors to Ten common statistical mistakes to watch out for when writing or reviewing a manuscript.

Sample-size estimation

- You should state whether an appropriate sample size was computed when the study was being designed
- You should state the statistical method of sample size computation and any required assumptions
- If no explicit power analysis was used, you should describe how you decided what sample (replicate) size (number) to use

Replicates

- You should report how often each experiment was performed
- You should include a definition of biological versus technical replication
- The data obtained should be provided and sufficient information should be provided to indicate the number of independent biological and/or technical replicates
- If you encountered any outliers, you should describe how these were handled
- Criteria for exclusion/inclusion of data should be clearly stated
- High-throughput sequence data should be uploaded before submission, with a private link for reviewers provided (these are available from both GEO and ArrayExpress)

Statistical reporting

- Statistical analysis methods should be described and justified

- Raw data should be presented in figures whenever informative to do so (typically when N per group is less than 10)
- For each experiment, you should identify the statistical tests used, exact values of N, definitions of center, methods of multiple test correction, and dispersion and precision measures (e.g., mean, median, SD, SEM, confidence intervals; and, for the major substantive results, a measure of effect size (e.g., Pearson's r, Cohen's d)
- Report exact p-values wherever possible alongside the summary statistics and 95% confidence intervals. These should be reported for all key questions and not only when the p-value is less than 0.05.

Group allocation

- Indicate how samples were allocated into experimental groups (in the case of clinical studies, please specify allocation to treatment method); if randomization was used, please also state if restricted randomization was applied
- Indicate if masking was used during group allocation, data collection and/or data analysis
